# Multiomics analysis revealed the mechanisms related to the enhancement of proliferation, metastasis and EGFR-TKI resistance in EGFR-mutant LUAD with ARID1A deficiency

**DOI:** 10.1186/s12964-023-01065-9

**Published:** 2023-03-03

**Authors:** Dantong Sun, Feiyue Feng, Fei Teng, Tongji Xie, Jinsong Wang, Puyuan Xing, Haili Qian, Junling Li

**Affiliations:** 1 Department of Medical Oncology, National Cancer Center/National Clinical Research Center for Cancer/Cancer Hospital, Chinese Academy of Medical Sciences and Peking Union Medical College, Beijing, 100021 China; 2 State Key Laboratory of Molecular Oncology, National Cancer Center/National Clinical Research Center for Cancer/Cancer Hospital, Chinese Academy of Medical Sciences and Peking Union Medical College, Beijing, 100021 China; 3 Department of Thoracic Surgery, National Cancer Center/National Clinical Research Center for Cancer/Cancer Hospital, Chinese Academy of Medical Sciences and Peking Union Medical College, Beijing, 100021 China

**Keywords:** *EGFR*-mutant LUAD, EGFR-TKI resistance, ARID1A, Multiomics analysis, Cell cycle

## Abstract

**Introduction:**

Dysregulated ARID1A expression is frequently detected in lung adenocarcinoma (LUAD) and mediates significant changes in cancer behaviors and a poor prognosis. ARID1A deficiency in LUAD enhances proliferation and metastasis, which could be induced by activation of the Akt signaling pathway. However, no further exploration of the mechanisms has been performed.

**Methods:**

Lentivirus was used for the establishment of the ARID1A knockdown (ARID1A-KD) cell line. MTS and migration/invasion assays were used to examine changes in cell behaviors. RNA-seq and proteomics methods were applied. ARID1A expression in tissue samples was determined by IHC. R software was used to construct a nomogram.

**Results:**

ARID1A KD significantly promoted the cell cycle and accelerated cell division. In addition, ARID1A KD increased the phosphorylation level of a series of oncogenic proteins, such as EGFR, ErbB2 and RAF1, activated the corresponding pathways and resulted in disease progression. In addition, the bypass activation of the ErbB pathway, the activation of the VEGF pathway and the expression level changes in epithelial–mesenchymal transformation biomarkers induced by ARID1A KD contributed to the insensitivity to EGFR-TKIs. The relationship between ARID1A and the sensitivity to EGFR-TKIs was also determined using tissue samples from LUAD patients.

**Conclusion:**

Loss of ARID1A expression influences the cell cycle, accelerates cell division, and promotes metastasis. *EGFR*-mutant LUAD patients with low ARID1A expression had poor overall survival. In addition, low ARID1A expression was associated with a poor prognosis in *EGFR*-mutant LUAD patients who received first-generation EGFR-TKI treatment.

**Video abstract**

**Supplementary Information:**

The online version contains supplementary material available at 10.1186/s12964-023-01065-9.

## Introduction

The high morbidity and mortality rates of lung cancer indicate that it has become a severe problem for public health worldwide. The 5-year overall survival (OS) rate is lower than 20% in China according to the latest report [1]. Lung adenocarcinoma (LUAD), which accounts for approximately 40–50% of non-small cell lung cancer (NSCLC) cases in Asian patients, is a common pathological type of NSCLC [1, 2]. Approximately 40% of Asian LUAD patients harbor driver mutations and could benefit from corresponding targeted therapy, such as tyrosine kinase inhibitors (TKIs) targeting sensitive mutations of epidermal growth factor receptor (EGFR) [3] or the rearrangement of anaplastic lymphoma kinase (ALK) [4]. Mutations were detected in exons 18 to 21 of the *EGFR* gene, while the majority of *EGFR* mutations are exon 19 deletions and exon 21 substitutions of leucine for arginine (L858R) [5, 6]. The presence of sensitive *EGFR* mutations prolonged LUAD patient OS based on treatment with EGFR-TKIs. However, patients might develop resistance to treatment and die due to the progression and metastasis of the disease.

The latest research confirmed the role of ARID1A, a member of the switch/sucrose nonfermenting (SWI/SNF) chromatin remodeling complex, which serves as a tumor suppressor in LUAD [7]. ARID1A is the key component of SWI/SNF complexes that can bind DNA in a non-sequence-specific manner and is involved in the processes of DNA repair and stabilization. Dysregulated ARID1A expression can be detected in multiple malignancies, including gastric cancer, bladder cancer, and especially LUAD, ARID1A loss is associated with the significant enhancement of tumor metastasis and a poor prognosis [7]. Loss of ARID1A in LUAD significantly enhances proliferation and metastasis, which could be induced by activation of the Akt signaling pathway [7]. However, no further exploration of the mechanisms was performed in the published study. In addition, given the essential role of the Akt pathway, the bypass activation of this pathway induced by ARID1A deficiency might contribute to resistance to EGFR-TKI treatment and should be further studied.

In this study, we established ARID1A knockdown (ARID1A-KD) LUAD cell lines harboring *EGFR* mutations and used multiomics analysis to explore the mechanisms that contributed to the enhancement of proliferation, migration and invasion in ARID1A-KD cells. This result suggested that ARID1A KD significantly promoted the cell cycle and led to the acceleration of tumor cell division. In addition, ARID1A KD increased the phosphorylation level of a series of oncogenic proteins, such as EGFR, ErbB2 and RAF1, activated the corresponding signaling pathways and finally resulted in the progression of the disease. In addition, the pathways activated by ARID1A KD provided strong evidence supporting resistance to EGFR-TKIs; the related changes included bypass activation of the ErbB pathway, activation of the VEGF pathway and expression level changes in epithelial–mesenchymal transformation (EMT) biomarkers. The relationship between ARID1A expression and sensitivity to EGFR-TKIs was also confirmed through tissue samples of LUAD patients.

## Materials and methods

### Bioinformatic analyses

In total, 13 datasets (4182 patients) of LUAD in the cBioPortal for Cancer Genomics [8, 9] were used to explore the role of *ARID1A* mutations in the OS of the disease. The PrognoScan (http://dna00.bio.kyutech.ac.jp/PrognoScan/index.html) database was used for the detection of ARID1A expression in influencing the prognosis of LUAD in different datasets. The dataset of *EGFR* mutant LUAD patients is available in The Cancer Genome Atlas (TCGA) (http://cancergenome.nih.gov/), and we also used this cohort to determine the role of ARID1A expression in the OS of stage III-IV *EGFR* mutant LUAD patients. The NCI-60 Cancer Cell Lines database was used for the evaluation of drug sensitivity influenced by the expression of ARID1A in NSCLC.

### Patients in the study

In total, 57 LUAD patients harboring sensitive *EGFR* mutations (exon 19 deletion or exon 21 L858R) who were admitted to our cancer center between August 2012 and October 2021 were enrolled in this study. Basic characteristics of the patients, including age, sex, *EGFR* mutation types and treatment information, were collected as listed in Table 1. All patients were diagnosed with stage IV LUAD in our department of pathology after hematoxylin and eosin (H&E) staining and immunohistochemical (IHC) evaluation. All patients had been administered first-line EGFR-TKI treatment. The disease status of each patient was evaluated using the standard Response Evaluation Criteria in Solid Tumors 1.1 (RECIST 1.1), and all tumors were staged according to the 2019 American Joint Committee on Cancer (AJCC) TNM staging system for lung cancer [10]. The Ethics Committee of Cancer Hospital Chinese Academy of Medical Sciences approved this study, and all investigations were carried out according to the rules of the Declaration of Helsinki. All experiments were carried out following the National Health and Family Planning Commission of the Professional Regulation Commission (PRC) guidelines.

**Table 1:** Information and survival analysis for involved EGFR mutant lung adenocarcinoma patients

Variables	Patients number (n = 57)	Univariate analysis	Multivariate analysis
*Gender*			
Male	23	0.7865	0.4540
Female	34		
*EGFR mutation types*			
Exon 19 deletion	24	0.6238	0.7540
Exon 21 L858R	33		
*Age*			
< = 60 years	28	0.4123	0.4020
> 60 years	29		
*ECOG*			
0–1	44	0.3488	0.0390*
2	13		
*EGFR-TKIs*			
Gefitinib	25	0.8944	0.8060
Erlotinib	17		
Icotinib	15		
*ARID1A expression*			
High expression	29	0.0015**	0.0040**
Low expression	28		

*P < 0.05; **P < 0.01

### IHC assay for ARID1A expression in *EGFR* mutant LUAD patients

IHC slides (samples before EGFR-TKI treatment) used for the evaluation of ARID1A expression were collected after obtaining informed consent from enrolled patients. Five micrometer thick sections were cut from paraffin-embedded tissues for subsequent IHC examination. Antigen retrieval was performed by boiling the slides in 10 mM citrate buffer (pH 6.0) for 10 min, followed by cooling at room temperature for 20 min. Each section was incubated at 4 °C with primary antibodies against ARID1A at appropriate concentrations (1:500) overnight. Two investigators independently evaluated the IHC slides. Five fields of each slide were selected for the evaluation of IHC scores. We followed a previously reported scoring method [7]. The intensity of staining was scored as 0 (no staining), 1 (weak), 2 (medium) and 3 (strong). Percentage scores were assigned as 0 (< 5%), 1 (5–25%), 2 (26–50%), 3 (51–75%) and 4 (76–100%). The final score of each slide was calculated as the average score of the 5 fields selected randomly and ranged from 0 to 12 (intensity score x percentage score). Specifically, low expression of ARID1A was defined as a final score less than 8 (IHC score < 8, 50th percentile of ARID1A IHC score, according to bioinformatic analysis). Antibody information is listed in Additional file 4: Table S1. As a positive control for ARID1A IHC examination, we used human kidney tissue, while normal human lung tissue was used as a negative control.

### Cell lines and construction of stable infectants [7]

The LUAD cell line with a sensitive *EGFR* mutation, HCC4006 (ATCC No.: CRL-2871), was purchased from the cell bank of the Chinese Academy of Sciences (Shanghai, China). Information for the STR Cell ID assay is depicted in Additional file 3. In addition, RNA samples of ARID1A KD and NC cells derived from the A549 cell line (kindly supplied by Prof. Helei Hou, from the Affiliated Hospital of Qingdao University), an *EGFR* wild-type human LUAD cell line, were also used to perform subsequent analysis to verify the findings in this study. The cell line was cultured in RPMI-1640 medium supplemented with 10% fetal bovine serum (FBS) and 1% P/S (100 IU/ml penicillin and 100 IU/ml streptomycin) in a 37 °C humidified atmosphere under 5% CO_2_. Lentiviral vectors encoding short hairpin RNAs (shRNAs) of ARID1A and a corresponding vector control (negative control [NC]) were purchased from GeneChem (Shanghai, China). We used helper solution (GeneChem, Shanghai, China) according to the manufacturer’s instructions to infect the cells, after which the infection efficiencies were verified by fluorescence microscopy. Cell counting (as a preliminary evaluation of the infection efficiency) revealed that over 90% of the cells expressed fluorescent protein, which was considered appropriate efficiency. Subsequently, changes in ARID1A expression were examined using Western blotting. Stably infected cell strains were selected for seven days and cultured with 2 μg/ml puromycin (Solarbio, Beijing, China). The sh-ARID1A and vector control sequences are listed in Additional file 4: Table S1.

### RNA-seq library construction and data analysis

We used TRIzol (Invitrogen, Carlsbad, CA) to extract the total RNA from the cultured cells. The total RNA was treated with RQ1 DNase (Promega) to remove the DNA. The quality and quantity of the purified RNA were determined by measuring the absorbance at 260 nm/280 nm (A260/A280) using a SmartSpec Plus spectrophotometer (Bio-Rad). Five micrograms of total RNA from each sample was used for RNA-seq library preparation. An Agilent 2100 Bioanalyzer was used for RNA quality assessment. NEBNext® Ultra™ Directional RNA Library Prep Kit for Illumina® was used for strand-specific library building. Then, sequencing by the synthesis method was used for Illumina sequencing. The libraries were sequenced via Illumina NovaSeq 6000, following the manufacturer’s instructions, by Novogene Co., Ltd. (Beijing, China). The uniquely mapped reads were obtained to calculate the read number and fragments per kilobase per million (FPKM) values for each gene. We used R studio software to analyze the differentially expressed genes (DEGs). To define the DEGs, log 2 (FoldChange) ≥ 1 and adjusted *P* ≤ 0.05 were set as the thresholds [11].

### Mass spectrometry (MS) analysis of total cellular protein

We performed label-free quantitative proteomics MS supported by Shanghai Bioprofile Technology Co., Ltd. The cell pellet was harvested, and the total protein was extracted using SDT cell lysis reagent. The digested peptides were desalted using Peptide Desalting Spin Columns and lyophilized under vacuum. Peptide concentrations were measured using a NanoDrop. When performing the MS for phosphorylated proteins, the peptide solution was lyophilized under a vacuum pump, and the phosphorylated peptides were enriched with an Fe-NTA Phosphopeptide Enrichment Kit (Thermo, A32992). Then, the enriched phosphorylated peptides were collected according to the kit procedure for mass spectrometry analysis. An appropriate amount of enriched peptides was taken from each sample for chromatographic separation using a nanoliter flow rate Easy nLC 1200 chromatographic system (Thermo Scientific). MSFragger 3.4 software was used to retrieve data from the UniProt Protein Data Bank (UniProt Homo sapiens (Human) [9606]-203800-202201.fasta). In this study, a log2 (FoldChange) ≥ 1.5 (total proteins) or 2 (phosphorylated proteins) and *P* ≤ 0.05 was considered to indicate significant differential expression of modifier sites.

### Western blot (WB) analysis [7]

Protein lysates of cells scratched from culture dishes were extracted using NP40 cell lysis reagent containing proteinase and phosphatase inhibitors (Beyotime) for 30 min on ice. Then, the cell lysates were centrifuged at 12,000 × g for 15 min at 4 °C, and the protein concentrations of the supernatants were determined using the BRADFORD method (Thermo Fisher). The supernatants were subsequently mixed with the appropriate volume of 5 × SDS loading buffer and heated at 100 °C for 10 min. Twenty milligrams of total protein from each sample was separated by SDS‒PAGE, transferred to 0.22 µm nitrocellulose membranes, blocked with 5% nonfat dry milk dissolved in PBST, and incubated overnight with primary antibodies at the appropriate dilutions (Additional file 4: **Table S1).** After being washed with PBST solution three times for 10 min each, the nitrocellulose membranes were incubated with HRP-conjugated secondary antibodies on a shaker for 1 h at room temperature. ECL reagent (Pierce, Rockford, IL, USA) was used to visualize the expression. ImageJ software was used to evaluate target protein expression.

### MTS assay

The cell line was seeded (5,000 cells/well) into two 96-well plates overnight to allow the cells to adhere. After 24 and 48 h of incubation, 100 µL of MTS solution (diluted with RPMI-1640 medium at 1:9) was added to each well, and the cells were incubated at 37 °C for another 1 h. Then, we measured the absorbance (A) at 490 nm on an ELISA plate reader.

### Cell migration and invasion assays [7]

Eight-micrometer pore Transwell compartments (Corning, NY) were used for cell migration and invasion assays. A total of 6 × 10^4^ cells were seeded into serum-free medium in the upper compartment and incubated for 48 h in a 37 °C humidified atmosphere under 5% CO_2_ for the migration assays. For the invasion assays, Matrigel (BD Biosciences, San Jose, CA) was added to each well, and 15 × 10^4^ cells were seeded and then incubated for 72 h. After incubation, the migrated cells in the lower chamber of the Transwell were fixed with formalin solution (Solarbio, 10% neutral buffered formalin) and stained with 0.5% crystal violet for 20 min at room temperature. The average number of migrated cells from five random views (at × 40 magnification) was calculated under a fluorescence orthotopic microscope (Nikon, Tokyo, Japan).

### Statistical analyses

The “rms” package of R software version 3.1.2 (The R Foundation for Statistical Computing, Vienna, Austria) was used to construct the nomogram in bootstrapping with 1000 resamples. The area under the curve (AUC) of the ROC curve was used to demonstrate the efficiency of ARID1A expression or the nomogram in predicting the progression-free survival (PFS) of *EGFR*-mutant LUAD patients who received EGFR-TKIs. Nonparametric tests were used, and *P* values were determined by two-tailed tests in this study. *P* < 0.05 was used to define statistical significance (*P* < 0.05: *; *P* < 0.01: **; *P* < 0.001: ***; *P* < 0.0001: ****) using GraphPad Prism 9.0 software (GraphPad, La Jolla, CA, USA). The GO BP, KEGG and REACTOME databases were used to run the enrichment analysis based on DEGs in RNA-seq or differentially expressed proteins (DEPs) in MS. The Database for Annotation, Visualization and Integrated Discovery (DAVID) (https://david.ncifcrf.gov/home.jsp) was used for functional annotation of DEGs and DEPs in this study. GraphPad Prism 9.0 software (GraphPad, La Jolla, CA, USA) and Bioinfo Intelligent Cloud (BIC) [12] were used for image plotting.

## Results

### ARID1A deficiency is associated with a poor prognosis in LUAD patients with or without sensitive *EGFR* mutations

**Fig. 1:**
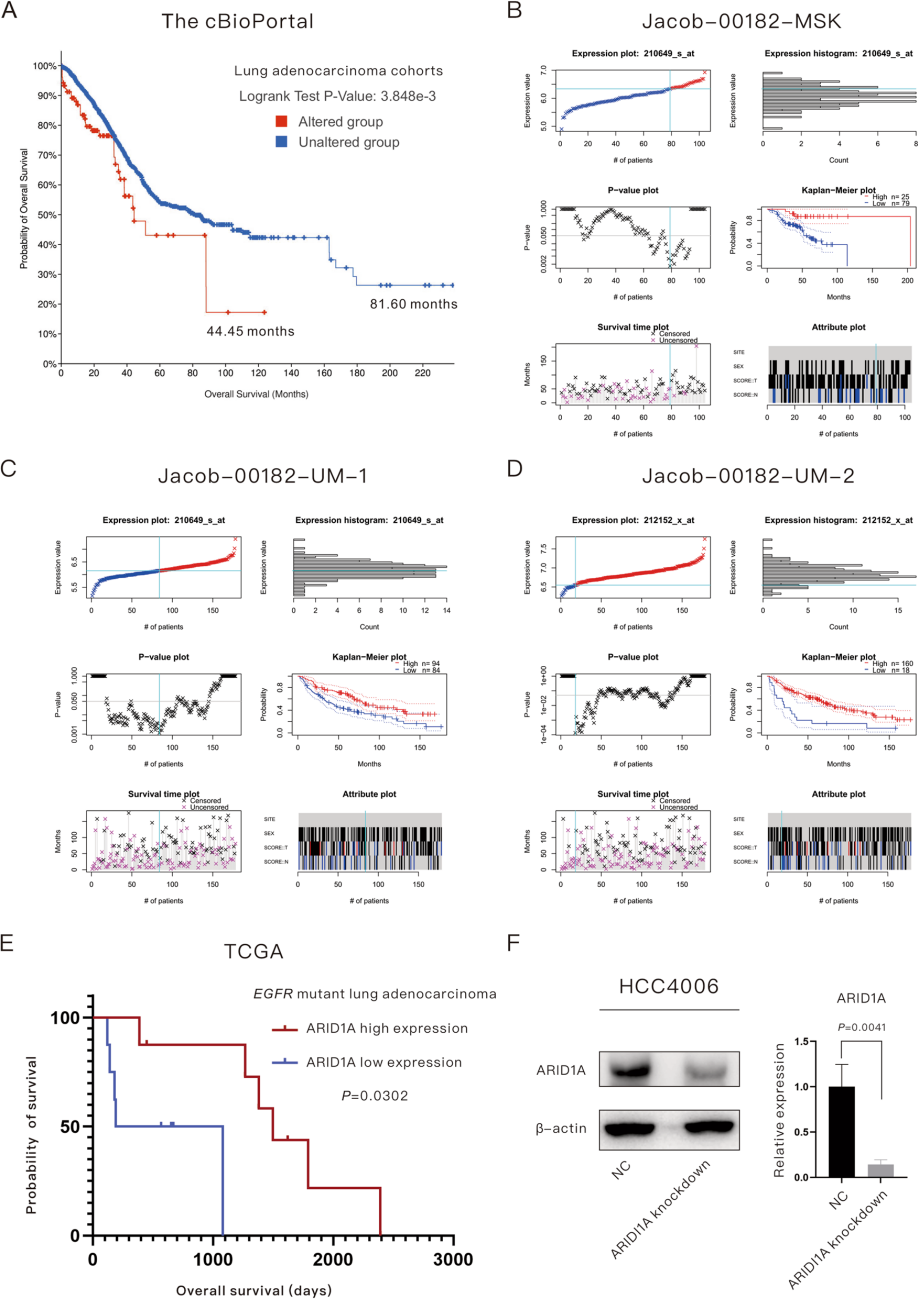
ARID1A serves as a protective factor in lung adenocarcinoma with or without ***EGFR*** mutations. **A** Lung adenocarcinoma patients harboring *ARID1A* mutations had poorer overall survival than the wild-type group. **B**–**D** Online database analysis revealed that low ARID1A expression was associated with poor overall survival in LUAD patients. **E** Low ARID1A expression was associated with a poor prognosis in *EGFR*-mutant lung adenocarcinoma patients in the TCGA database. **F** Verification of the relative expression of target proteins after the construction of ARID1A knockdown *EGFR*-mutant lung adenocarcinoma cell lines

A total of 228 patients (6%) among all 13 LUAD datasets in the cBioPortal database harbored *ARID1A* mutations, and patients harboring *ARID1A* mutations had poorer OS than the wild-type group (44.45 months versus 81.60 months, *P* = 0.0038), as shown in Fig. 1A. The majority of ARID1A mutations are inactivating and lead to expression loss according to previous research [7, 13];then, the function of ARID1A expression was studied. In the current study, low ARID1A expression was found to be related to poor OS in LUAD patients according to the Jacob-00182-MSK dataset (univariate analysis: *P* = 0.0383; multivariate analysis: *P* = 0.0177; Fig. 1B), Jacob-00182-UM dataset for 210649_s_at (univariate analysis: *P* = 0.0288; multivariate analysis: *P* = 0.0019; Fig. 1C) and Jacob-00182-UM dataset 212152_x_at (univariate analysis: *P* = 0.0045; multivariate analysis: *P* = 0.0109; Fig. 1D) in the PrognoScan database. To determine the role of ARID1A expression in the OS of EGFR-mutant LUAD patients, we performed survival analysis based on a cohort of 16 stage III-IV EGFR-mutant LUAD patients derived from the TCGA database, as displayed in Fig. 1E. EGFR-mutant LUAD patients with low ARID1A expression had a poorer OS than the high expression group (635 days versus 1498 days, *P* = 0.0302). In addition, we explored the role of ARID1B, whose function is close to ARID1A, in the prognosis of EGFR-mutant LUAD patients. However, ARID1B had no prognostic effect on the patients (*P* = 0.6632).

### Multiomics analysis revealed the mechanisms related to the phenotypic changes in *EGFR-*mutant LUAD induced by ARID1A KD

**Fig. 2:**
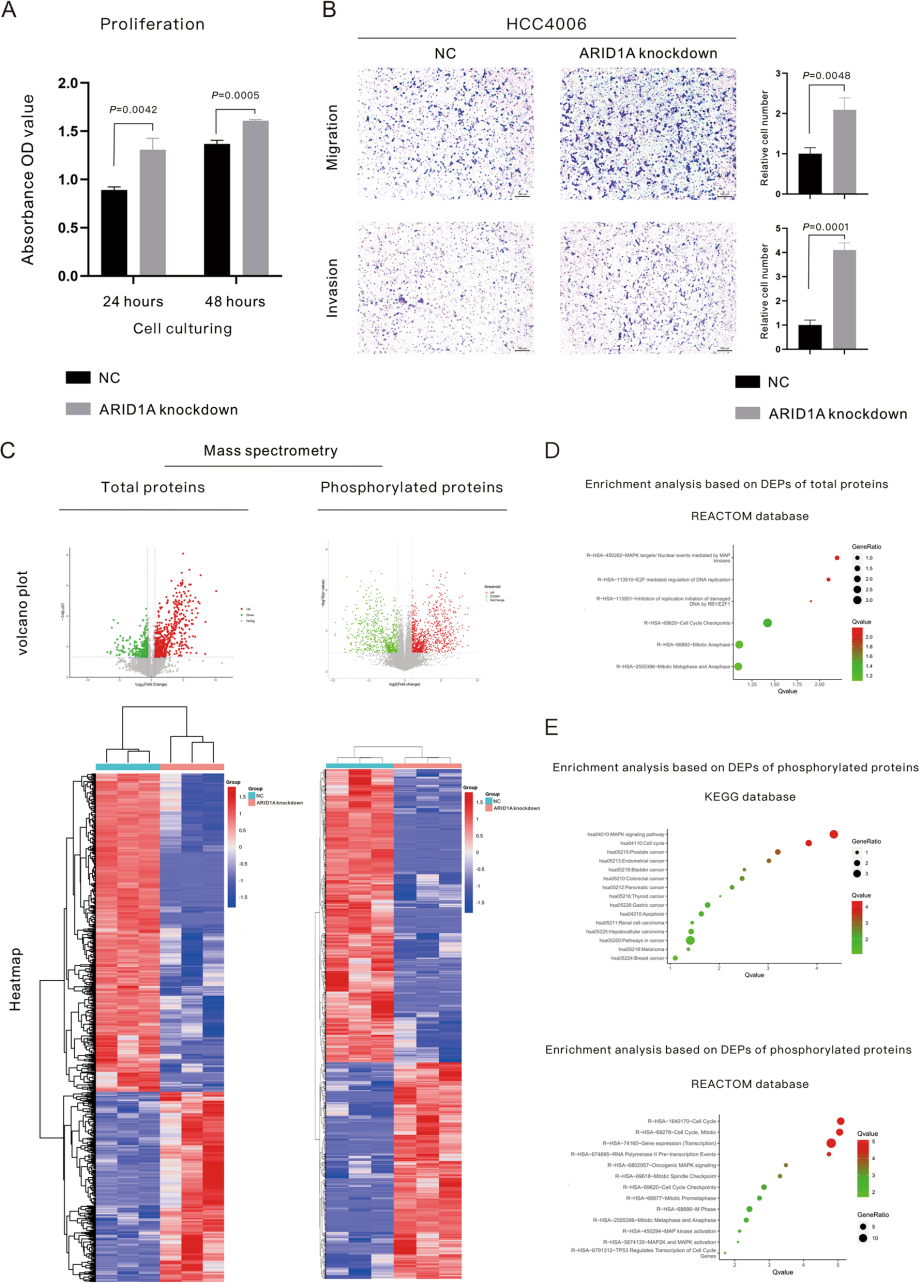
ARID1A knockdown significantly enhanced the proliferation, migration and invasion of EGFR mutant lung adenocarcinoma cells. **A** Proliferative examination using MTS experiments; **B** Migration and invasion examination using Transwell assays. **C** Volcano plots and heatmap of differentially expressed proteins revealed by mass spectrometry. **D**–**E** Enrichment analysis for differentially expressed proteins revealed by mass spectrometry for total proteins and phosphorylated proteins

Given the clinical evidence that low ARID1A expression is associated with the poor prognosis of *EGFR-*mutant LUAD, we then performed a subsequent multiomics analysis to explore the concrete mechanisms for this relationship. The ARID1A-KD cell line was constructed using the human *EGFR* mutant LUAD cell line (HCC4006), and the evaluation of the knockdown effect is displayed in Fig. 1F. The phenotypic changes in the proliferation, migration and invasion of ARID1A-KD cells compared with NC cells are displayed in Fig. 2A, B. The 490 nm OD value of live cells in the ARID1A KD group was higher than that in the NC group after cell incubation for 24 h (*P* = 0.0042) and 48 h (*P* = 0.0005), which suggested the enhancement of tumor cell proliferation in ARID1A-KD cells (Fig. 2A). Using transwell experiments (Fig. 2B), the changes in the migration and invasion abilities of tumor cells were elucidated. ARID1A KD significantly elevated the migration (*P* = 0.0048) and invasion (*P* = 0.0001) abilities of *EGFR*-mutant LUAD cells.

**Fig. 3:**
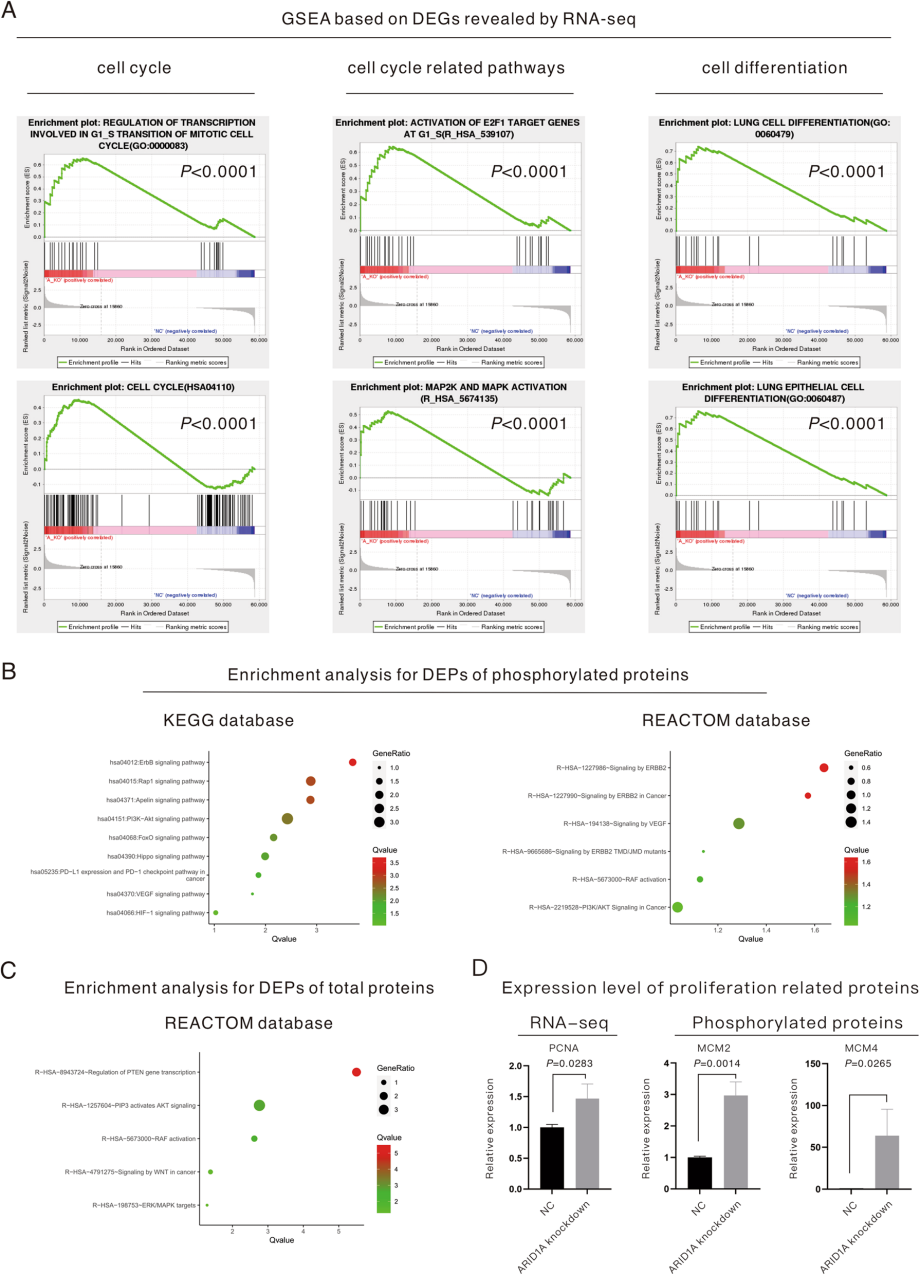
Multiomics analysis revealed the underlying mechanisms of the enhancement of proliferation and metastasis induced by ARID1A knockdown. **A** GSEA based on differentially expressed genes revealed by RNA-seq sequencing. **B**–**C**. Enrichment analysis based on differentially expressed proteins revealed by mass spectrometry for total proteins and phosphorylated proteins. **D** Relative expression level of proliferative biomarkers revealed by RNA-seq sequencing and mass spectrometry

To explore the underlying mechanisms related to the phenotype changes in ARID1A-KD cells, RNA-seq sequencing and MS for total proteins and phosphorylated proteins were performed (the volcano plots and heatmap for DEPs revealed by MS are displayed in Fig. 2C). For the underlying mechanisms for cell proliferation enhancement, MS for total proteins (Fig. 2D) demonstrated the activation of the RB-E2F pathway and the level change of DNA replication and the cell cycle based on the REACTOME database. Moreover, MS for phosphorylated proteins (Fig. 2E) revealed activation of the MAPK pathway and changes in the cell cycle based on the KEGG and REACTOME databases. In addition, enrichment analysis based on the KEGG database (Fig. 2E) indicated that ARID1A KD was tightly associated with pathways related to a variety of malignancies, including gynecological malignancies and gastrointestinal malignancies. In addition, the enrichment analysis based on DEGs revealed by RNA-seq sequencing (Fig. 3A) also supported that the activation of the MAPK and RB-E2F pathways and the downstream level change in the cell cycle were responsible for the proliferative acceleration in ARID1A-KD cells. The enrichment analysis for RNA-seq also showed that ARID1A KD influenced the differentiation of lung cells or lung epithelial cells (Fig. 3A). On the other hand, the enrichment analysis based on MS for phosphorylated proteins (Fig. 3B) and total proteins (Fig. 3C) indicated a series of pathways related to tumor development and metastasis, including the ErbB pathway, RAF pathway, VEGF pathway, HIF-1 pathway and so on. Given this evidence, we performed a subsequent analysis to verify the underlying mechanisms.

### ARID1A KD promoted the cell cycle of* EGFR*-mutant LUAD cells and accelerated proliferation

**Fig. 4:**
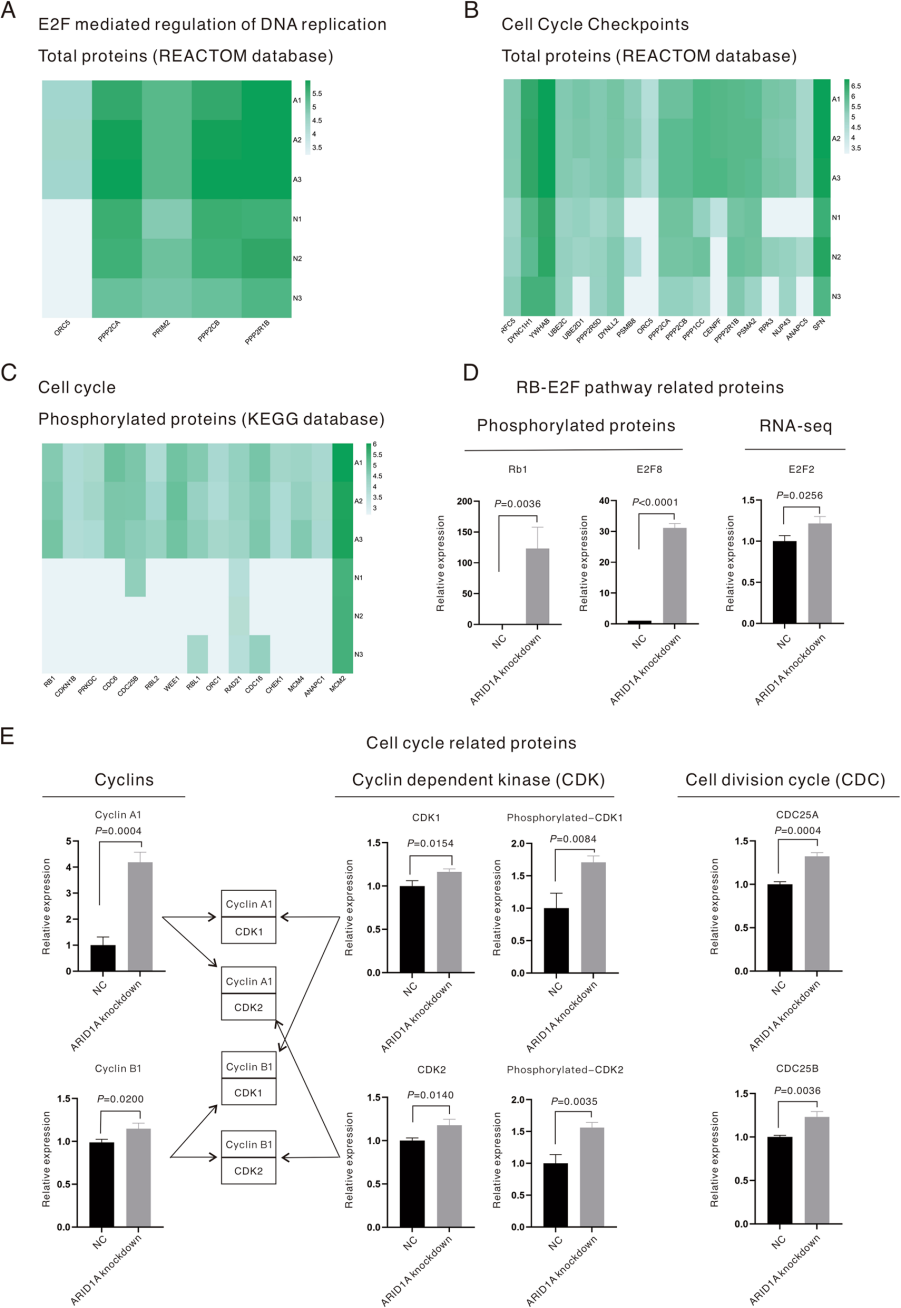
ARID1A knockdown promoted the cell cycle of EGFR-mutant lung adenocarcinoma and increased the cell proliferation rate**.** **A**–**C** Proteins enriched in the cell cycle-related pathways revealed by mass spectrometry (all listed proteins were found to be upregulated in ARID1A knockdown cells with statistical significance). **D** Relative expression levels of RB-E2F pathway-related proteins revealed by RNA-seq sequencing and mass spectrometry. **E** Relative expression levels of cell cycle proteins, including cyclins, cyclin-dependent kinase and cell division cycle proteins, revealed by mass spectrometry

In addition to the change in the proliferation of *EGFR*-mutant LUAD cells induced by ARID1A KD (Fig. 2A), the expression levels of proliferative biomarkers were also examined through RNA-seq sequencing and MS (Fig. 3D). The results demonstrated that proliferation-related biomarkers, including PCNA (*P* = 0.0283), phosphorylated MCM2 (*P* = 0.0014) and phosphorylated MCM4 (*P* = 0.0265), were all upregulated in ARID1A-KD cells. Figure 4A–C displays the enriched pathways in ARID1A-KD cells based on the DEPs revealed by MS, and all the listed genes were found to be significantly upregulated in ARID1A-KD cells versus control cells. The RB-E2F pathway was activated in ARID1A-KD cells, which started with the elevation of the phosphorylation level of RB1 (*P* = 0.0036, Fig. 4D) and was followed by the activation of E2F family members. In ARID1A-KD cells, E2F2 (*P* = 0.0256, Fig. 4D) and phosphorylated E2F8 (*P* < 0.0001, Fig. 4D) were upregulated, and the other proteins related to this pathway are all displayed in Fig. 4A. It is well established that the phosphorylation of RB1 releases E2F family members and influences the cell cycle. The other upregulated proteins were cell cycle checkpoints (total proteins, REACTOME database, Fig. 4B) and cell cycle-related proteins (phosphorylated proteins, KEGG database, Fig. 4C). The expression of proteins related to the cell cycle, including cyclins, cyclin-dependent kinase (CDK) and the cell division cycle (CDC), was then evaluated through RNA-seq and MS, as displayed in Fig. 4E. The expression of cyclins, including cyclin A1 (*P* = 0.0004, Fig. 4E) and cyclin D3 (*P* = 0.0028, Fig. 4E), was upregulated in ARID1A-KD cells. As for CDKs, both CDK1 (*P* = 0.0154, Fig. 4E) and CDK2 (*P* = 0.0140, Fig. 4E), as well as phosphorylated CDK1 (*P* = 0.0084, Fig. 4E) and phosphorylated CDK2 (*P* = 0.0035, Fig. 4E), were upregulated in ARID1A-KD cells. Phosphorylated WEE1 (*P* = 0.0006, Fig. 4C) was upregulated in ARID1A-KD cells and is responsible for the phosphorylation of CDKs at the Thr14 and Tyr15 sites. CDC proteins, such as the CDC25 family proteins, dephosphorylate Thr14 and Tyr15 of CDK and endow CDKs with kinase activity. In the current study, CDC25 family members, including CDC25A (*P* = 0.0004, Fig. 4E) and CDC25B (*P* = 0.0036, Fig. 4E), were found to be upregulated in ARID1A-KD cells. According to this evidence, the cell cycle and division of ARID1A-KD cells were accelerated to improve proliferation.

### The enhancement of migration, invasion or metastasis in ARID1A-KD *EGFR*-mutant LUAD involves the cooperation of multiple factors and mechanisms

**Fig. 5:**
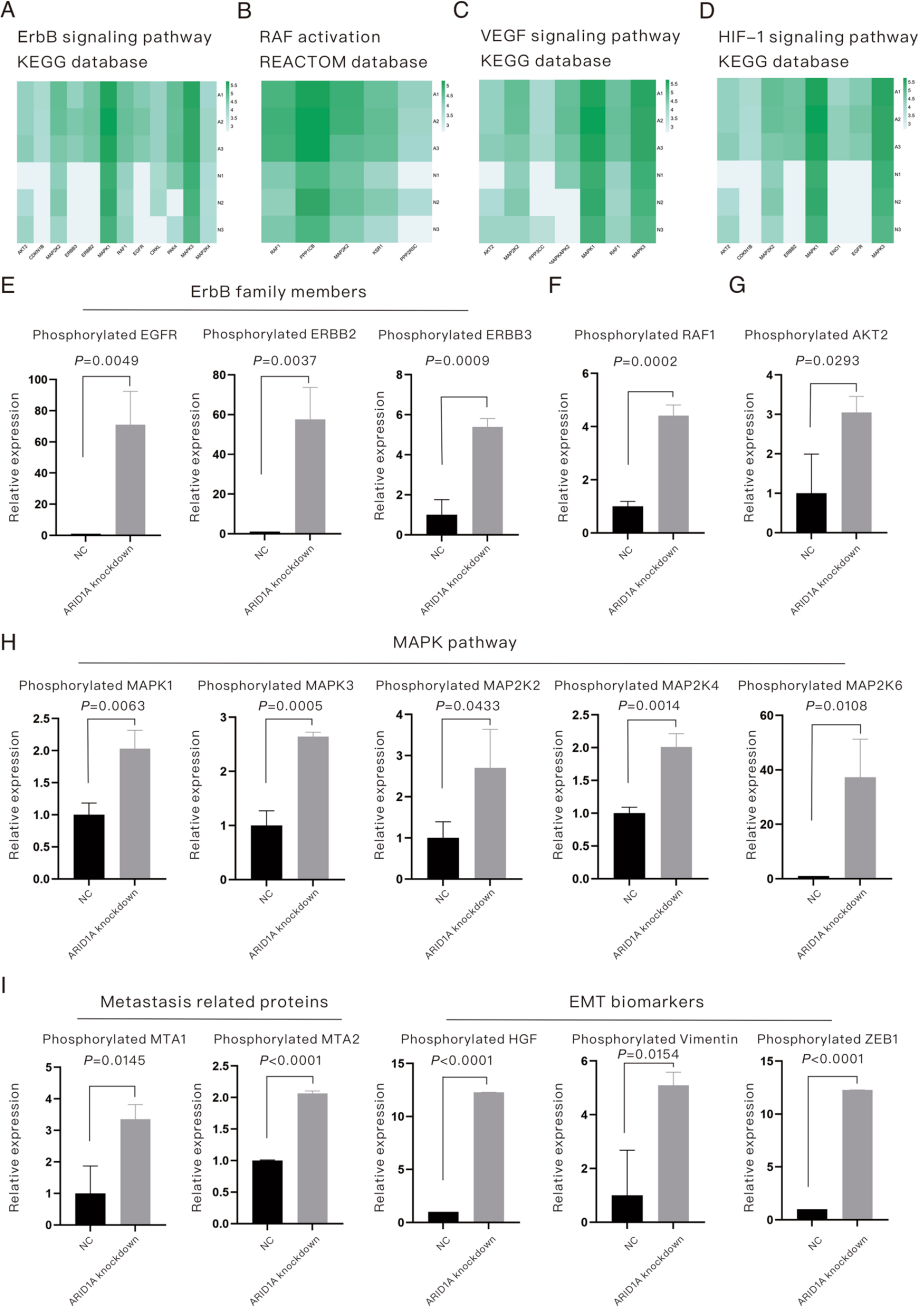
The enhancement of metastasis in ARID1A knockdown EGFR-mutant lung adenocarcinoma is a process involving multiple factors and multiple mechanisms. **A**–**D** Proteins enriched in metastasis-related pathways revealed by mass spectrometry (all listed proteins were upregulated in ARID1A knockdown cells with statistical significance). **E** Relative expression levels of phosphorylated ErbB family members. **F** Relative expression level of phosphorylated RAF1. **G** Relative expression level of phosphorylated AKT2. **H** Relative expression levels of phosphorylated proteins belonging to the MAPK pathway. **I** Relative expression levels of phosphorylated metastasis-related proteins and EMT biomarkers

As described in the published study [7] and the current study (Fig. 2B), ARID1A KD promotes the migration, invasion and metastasis of EGFR-mutant LUAD cells. In addition, enrichment analysis (Fig. 3B, C) indicated the underlying pathways related to this phenotype change. Here, we proposed that the promotion of migration, invasion and metastasis in EGFR-mutant LUAD cells induced by ARID1A KD is a cooperative process involving multiple factors and multiple mechanisms and should be discussed in detail. Figure 5A–D (all listed phosphorylated proteins were found to be upregulated in ARID1A-KD cells with statistical significance) displays the enriched pathways in ARID1A-KD cells based on the KEGG and REACTOME databases, and these pathways are associated with tumor progression and metastasis. Therefore, we subsequently explored the related mechanisms. First, members of the ErbB family, including phosphorylated EGFR (*P* = 0.0049), phosphorylated ERBB2 (*P* = 0.0037) and phosphorylated ERBB3 (*P* = 0.0009), were all upregulated in ARID1A-KD cells, as shown in Fig. 5E. We also verified the activation of ErbB family members, as shown in Additional file 1: Figure S1. Phosphorylated RAF1 (*P* = 0.0002, Fig. 5F) and AKT2 (*P* = 0.0293, Fig. 5G) were also upregulated in ARID1A-KD cells. The activation of the MAPK pathway was also revealed in this study, as displayed in Fig. 5H. Phosphorylated MAPK1 (*P* = 0.0063), MAPK3 (*P* = 0.0005), MAP2K2 (*P* = 0.0433), MAP2K4 (*P* = 0.0014) and MAP2K6 (*P* = 0.0108) were all upregulated in ARID1A-KD cells. To further confirm the role of ARID1A KD in regulating these signaling pathways, we performed Western blotting assays in HCC4006 and NCI-H1299 cell lines, as shown in Additional file 1: Figure S1. It suggested that ARID1A-KD significantly elevated the phosphorylated expression levels of MAPK, Akt, EGFR, HER2 and HER4. Besides, ARID1A-KD elevated the total expression level of HER3, and therefore the upregulation of phosphorylated HER3 (no significant elevation of phosphorylate form/total form ratio). Biomarkers for metastasis, including metastasis-related proteins and EMT markers, were also evaluated in this study, as shown in Fig. 5I, and phosphorylated MTA1 (*P* = 0.0145), MTA2 (*P* < 0.0001), HGF (*P* < 0.0001), Vimentin (*P* = 0.0154) and ZEB1 (*P* < 0.0001) were all upregulated in ARID1A-KD cells. Briefly, ARID1A KD activates a series of pathways to participate in the migration, invasion and metastasis of *EGFR*-mutant LUAD.

### ARID1A expression serves as a novel biomarker for resistance to first-generation EGFR-TKIs in *EGFR*-mutant LUAD patients

**Fig. 6:**
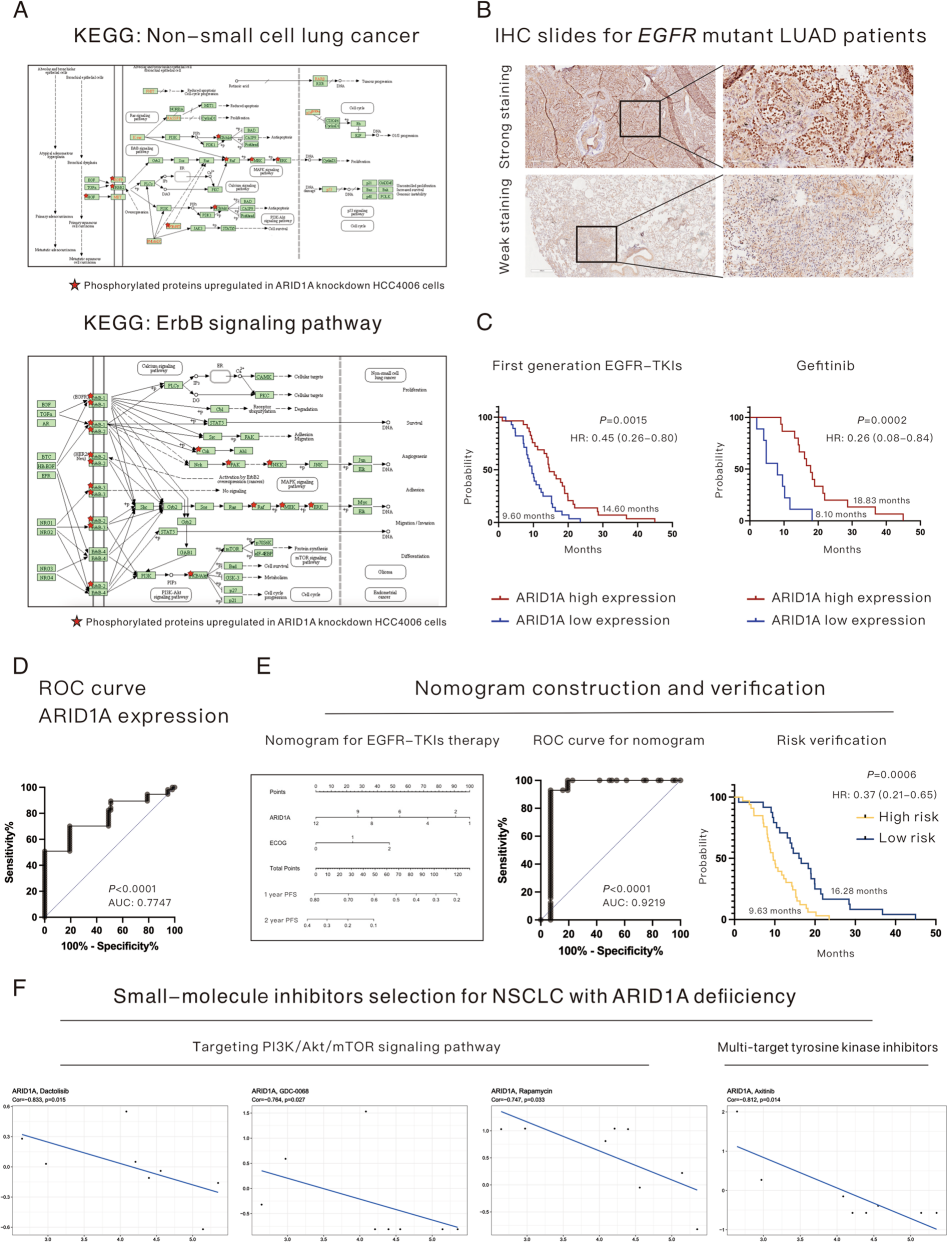
ARID1A expression serves as a novel biomarker for resistance to first-generation EGFR-TKIs in EGFR-mutant lung adenocarcinoma patients. **A** Underlying mechanisms based on differentially expressed proteins revealed by mass spectrometry for phosphorylated proteins, which might be associated with resistance to EGFR-TKIs. **B** Representative images of IHC staining of ARID1A in *EGFR*-mutant lung adenocarcinoma. **C** Progression-free survival after treatment with first-generation EGFR-TKIs in all enrolled patients and patients who received gefitinib (divided by ARID1A expression). **D** ROC curve for ARID1A expression in predicting the progression-free survival of patients treated with first-generation EGFR-TKIs. **E** The construction and verification of a nomogram based on ARID1A expression and ECOG score for *EGFR*-mutant lung adenocarcinoma patients who received first-generation EGFR-TKIs. **F** Drug selection based on the NCI-60 database for non-small cell lung cancer patients with ARID1A deficiency

Multiomics analysis revealed that multiple mechanisms participated in the enhancement of proliferation and metastasis in ARID1A-KD *EGFR* mutant LUAD and could be summarized as shown in Fig. 6A according to the KEGG database. The NSCLC-related pathways and the bypass activation of the ErbB pathway strongly indicated that ARID1A KD might result in resistance to EGFR-TKIs. To verify the role of ARID1A in EGFR-TKI treatment, 57 *EGFR* mutant LUAD patients were enrolled. The cohort of patients was divided into an ARID1A high expression group and a low expression group according to the median IHC score. Representative images of strong and weak ARID1A staining are shown in Fig. 6B. As shown in Fig. 6C, patients in the ARID1A high expression group had a longer PFS time than those in the low expression group (14.60 months versus 9.60 months, *P* = 0.0015) after the administration of first-generation EGFR-TKIs, and the hazard ratio (HR) was 0.45 (95% confidence interval [95% CI]: 0.26–0.80). In this study, a total of 3 kinds of first-generation EGFR-TKIs, including gefitinib, erlotinib and icotinib, were administered to the patients, as shown in Table 1. Through survival analysis, we found that ARID1A expression could be a robust biomarker in patients who received gefitinib (low expression group versus high expression group: 8.10 months versus 18.83 months, *P* = 0.0002, Fig. 6C), and the HR was 0.26 (95% CI: 0.08–0.84) but not in patients who received erlotinib (low expression group versus high expression group: 8.90 months versus 12.75 months, *P* = 0.1647) or icotinib (low expression group versus high expression group: 11.80 months versus 12.92 months, *P* = 0.7113). Even so, treatment benefits could still be detected in patients with high ARID1A expression who received erlotinib or icotinib. The ROC curve for ARID1A expression in EGFR-TKI treatment, as shown in Fig. 6D**,** demonstrated that ARID1A expression is a relatively robust biomarker for PFS of EGFR-TKIs (AUC: 0.7747, *P* < 0.0001); however, we established a prognostic nomogram based on the Cox multivariate regression model to improve the efficiency of ARID1A in identifying patients who would benefit more from EGFR-TKI treatment (Fig. 6E). This demonstrated that ARID1A expression combined with the ECOG score could better predict the PFS of EGFR-mutant LUAD patients who received first-generation EGFR-TKIs. The ROC curve of the nomogram (Fig. 6E) revealed that the prognostic model had better efficiency (AUC: 0.9219, *P* < 0.0001) than a single variable (ARID1A expression), as shown in Fig. 6D. According to the novel nomogram, the enrolled patients were scored and divided into a high-risk group and a low-risk group (cutoff: 52.86). Survival analysis demonstrated that the risk score was efficient in predicting the PFS of patients with EGFR-TKI treatment (high-risk group versus low-risk group: 9.63 months versus 16.28 months, *P* = 0.0006, Fig. 6E), and the HR was 0.37 (95% CI: 0.21–0.65). In addition, we analyzed the relationship between ARID1A expression and the EGFR 20 exon T790M mutation, which serves as the common mechanism for EGFR-TKI resistance. The results demonstrated that 42.9% (16/36) of patients harboring T790M mutations had low expression of ARID1A compared with 57.1% (12/21) of patients without T790M mutations, which indicated that ARID1A deficiency could participate more in the development of EGFR-TKI resistance in T790M-negative patients. Additionally, we assessed the IC50 of osimertinib, which is the first-line treatment choice for advanced EGFR-mutant LUAD, in the HCC4006 cell line and found that ARID1A KD significantly increased the IC50 of osimertinib (6.2 µM versus 13.2 µM, *P* = 0.0023).

To explore potential drugs to overcome the disease progression of NSCLC patients with ARID1A deficiency, the NCI-60 database was used, and a series of small-molecule inhibitors were found to be effective (Fig. 6F). The results demonstrated that inhibitors targeting the PI3K/Akt/mTOR pathway, including dactolisib (pan-class I PI3K & mTOR inhibitor, Cor = −0.833, *P* = 0.015), GDC-0068 (pan-Akt inhibitor, Cor = −0.764, *P* = 0.027) and rapamycin (mTOR inhibitor, Cor = −0.747, *P* = 0.033), might be useful for the treatment of NSCLC patients with low ARID1A expression. In addition, axitinib (multitarget inhibitor with targets including VEGFR1, VEGFR2, VEGFR3 and PDGFR-β, Cor = −0.812, *P* = 0.014) could also be a choice for treatment.

## Discussion

Mutations in *ARID1A* are diverse and have been discovered in a variety of malignancies, including gynecological carcinoma [14], urothelial carcinoma [15], gastric cancer [16] and lung cancer [7]. The majority of *ARID1A* mutations are inactivating and lead to the loss of ARID1A expression [7, 13]. Previous research reported that loss of intact BAF, and especially the functional loss of ARID1A, endows cells with cancerous functions and leads to a poor prognosis for multiple types of cancer. Specifically, in LUAD, loss of ARID1A expression promotes tumor proliferation and metastasis in vitro and in vivo [7]. The results of the current study provide clear support for the role of ARID1A expression loss in enhancing the proliferation and metastasis of LUAD, which is consistent with previous research. Mechanistically, previous studies proved that ARID1A expression loss activates the PI3K/Akt pathway to induce oncogenic effects in multiple malignancies, including gastric cancer [16], gynecological cancers [17–19] and lung cancer [7]. However, no further exploration of upstream or downstream mechanisms related to ARID1A expression loss was performed. In this study, we shed new light on the role of ARID1A expression loss in altering multiple oncogenic pathways to orchestrate the behaviors of LUAD. The results suggested that ARID1A expression loss activates a series of receptor tyrosine kinases (RTKs), such as EGFR, ERBB2 and ERBB3, which belong to the ErbB family, and induced multiple mechanisms to enhance proliferation and metastasis. On the one hand, ARID1A expression loss leads to the phosphorylation of RB1, releases the inhibition of the E2F family and initiates preparation for cell division. Loss of ARID1A expression also leads to the upregulation of cell cycle-related proteins, including cyclins, CDKs and CDC proteins, increases the activity of CDKs and accelerates the cell division process. On the other hand, ARID1A expression loss triggers a variety of pathways related to tumor progression and metastasis, including the ErbB pathway, VEGF pathway, HIF-1 pathway and RAF1 pathway, to participate in metastasis. In addition, ARID1A expression loss could endow tumor cells with an EMT phenotype and metastatic ability [20]. In summary, ARID1A serves as the essential tumor suppressor in *EGFR*-mutant LUAD, while loss of ARID1A expression leads to the extensive activation of oncogenic pathways and enables tumor cells to acquire a more aggressive phenotype, finally resulting in the progression of the disease. To further expand our findings, we performed RNA-seq sequencing in an *EGFR* wild-type LUAD cell line (A549), and the sequencing results confirmed the role of ARID1A in LUAD, as shown in Additional file 2: Figure S2. This result suggested that ARID1A expression loss could significantly alter the process of cell division by influencing the cell cycle of tumor cells in *EGFR* wild-type LUAD and activate a series of pathways, including the oncogenic MAPK pathway and ERBB2 pathway, to initiate and accelerate the development of the disease.

Small molecular inhibitors or monoclonal antibodies for EGFR signaling have been widely used for EGFR signaling-dependent malignancies [21, 22]. A previous review [21] proposed the potential role of ARID1A as a novel biomarker for the response to EGFR-TKIs. *ARID1A* mutations or expression loss might be regulators of multiple pathways related to resistance to EGFR-TKIs. In addition, previous research has also suggested that *ARID1A* mutations are associated with shorter PFS after EGFR-TKI treatment [23]. In the current study, the results led to a similar conclusion that *EGFR*-mutant LUAD patients with low ARID1A expression had worse PFS than those with high ARID1A expression. First, multiomics analysis revealed various mechanisms that contributed to resistance to EGFR-TKIs, including bypass activation of the ErbB pathway and VEGF pathway. Therefore, a cohort of *EGFR* mutant LUAD patients was enrolled for the analysis. These results suggest that ARID1A expression serves as a biomarker for the response to EGFR-TKI treatment with satisfactory efficiency. The novel nomogram based on ARID1A expression and ECOG score exhibited robust efficiency in predicting the PFS of patients after first-generation EGFR-TKI treatment and thus could be used in risk evaluation before EGFR-TKI administration. Second, we proposed a series of small-molecule inhibitors that might be potential therapeutics for the treatment of NSCLC with ARID1A deficiency. Our results demonstrated that drugs targeting the PI3K/Akt/mTOR pathway serve as promising treatments for patients, as well as multitarget TKIs. Admittedly, several limitations exist in this study. Only one cell line was used in this study, and the results need to be further validated in expanded experiments. In addition, in vivo experiments are needed to further verify the results. In addition, whether ARID1A expression loss could induce resistance to second- and third-generation EGFR-TKIs requires further study.

## Conclusion

ARID1A serves as a tumor suppressor in *EGFR*-mutant LUAD. Loss of ARID1A expression activates a series of oncogenic pathways initiated by the phosphorylation of RTKs, which influences the cell cycle, accelerates cell division and promotes metastasis. *EGFR*-mutant LUAD patients with low ARID1A expression have a poor prognosis. In addition, low ARID1A expression is associated with a poor response to first-generation EGFR-TKIs in *EGFR*-mutant LUAD patients.

## Supplementary Information

**Additional file 1** Figure S1. Expression evaluation of targeted proteins in HCC4006 and NCI-H1299 cell lines using Western blotting.

**Additional file 2** Figure S2. Enrichment analysis based on differentially expressed genes revealed by RNA-seq sequencing of the A549 cell line.

**Additional file 3.** Cell Line Authentication Service. STR ProfilingReport.

**Additional file 4. Table S1**. Antibodies and lentivirus sequences.

## Data Availability

All data and materials are mentioned in this article and can also be requested by email (sundantongerik@163.com).

## Abbreviations

OS: Overall survival
LUAD: Lung adenocarcinoma
NSCLC: Non-small cell lung cancer
TKIs: Tyrosine kinase inhibitors,
EGFR: Epidermal growth factor receptor
ALK: Anaplastic lymphoma kinase
SWI/SNF: Switch/sucrose nonfermenting
ARID1A KD: ARID1A knockdown
EMT: Epithelial interstitial transformation
TCGA: The Cancer Genome Atlas
H&E: Hematoxylin and eosin
IHC: Immunohistochemistry
RECIST 1.1: Response Evaluation Criteria in Solid Tumors 1.1
AJCC: American Joint Committee on Cancer
FBS: Fetal bovine serum
FPKM: Fragments per kilobase and per million
DEGs: Differentially expressed genes
PFS: Progression-free survival
DEPs: Differentially expressed proteins
BIC: Bioinfo Intelligent Cloud
CDK: Cyclin-dependent kinase
CDC: Cell division cycle
