# Supplementary figures and images for: Multiomics analysis revealed the mechanisms related to the enhancement of proliferation, metastasis and EGFR-TKI resistance in EGFR-mutant LUAD with ARID1A deficiency
Source: Cell Commun Signal. 2023 Mar 3;21:48. doi: 10.1186/s12964-023-01065-9 (PMC9985251; doi:10.1186/s12964-023-01065-9)

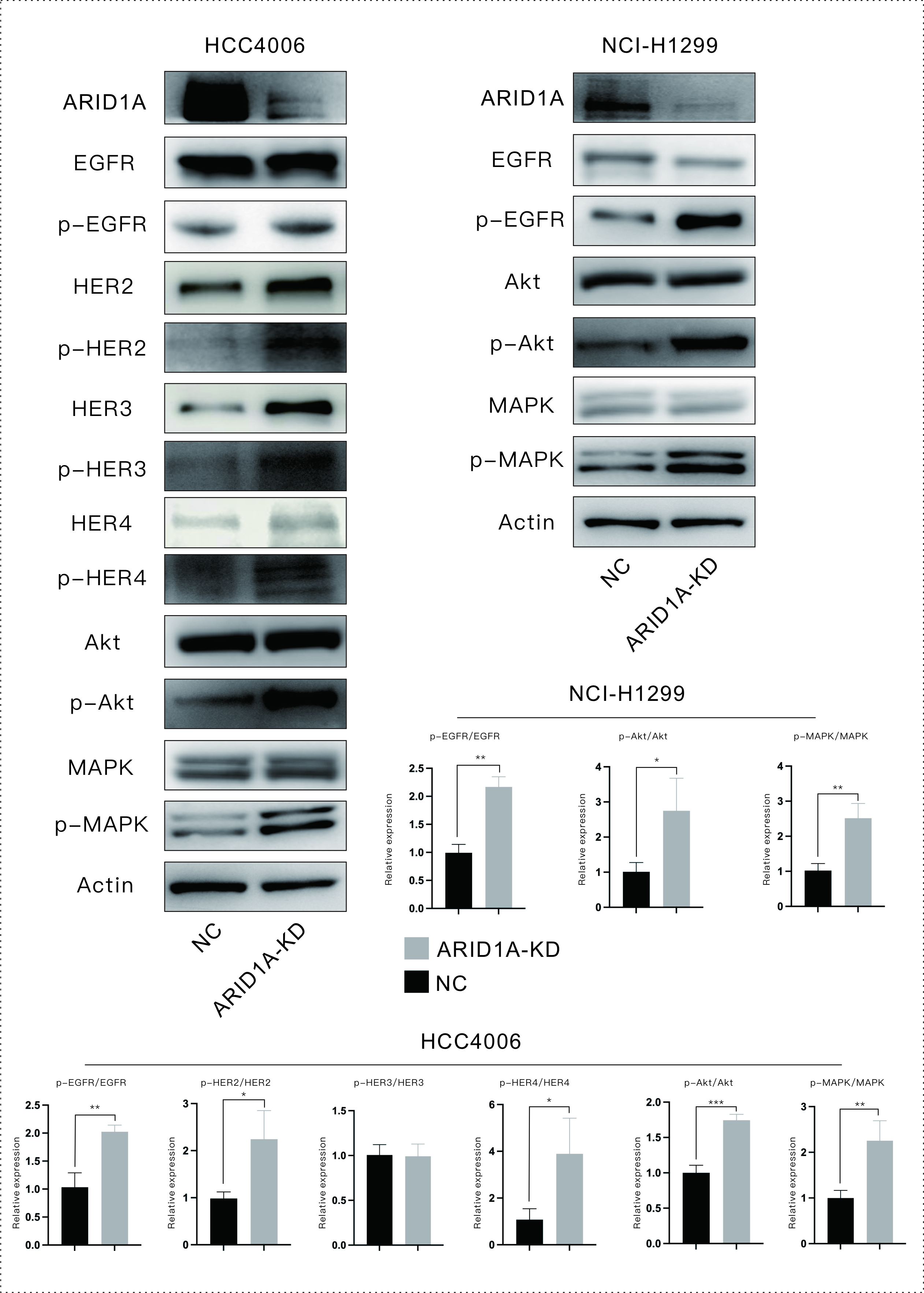

Supplement: Supplementary file 2 — Additional file 1 Figure S1. Expression evaluation of targeted proteins in HCC4006 and NCI-H1299 cell lines using Western blotting. [file 12964_2023_1065_MOESM2_ESM.jpg]

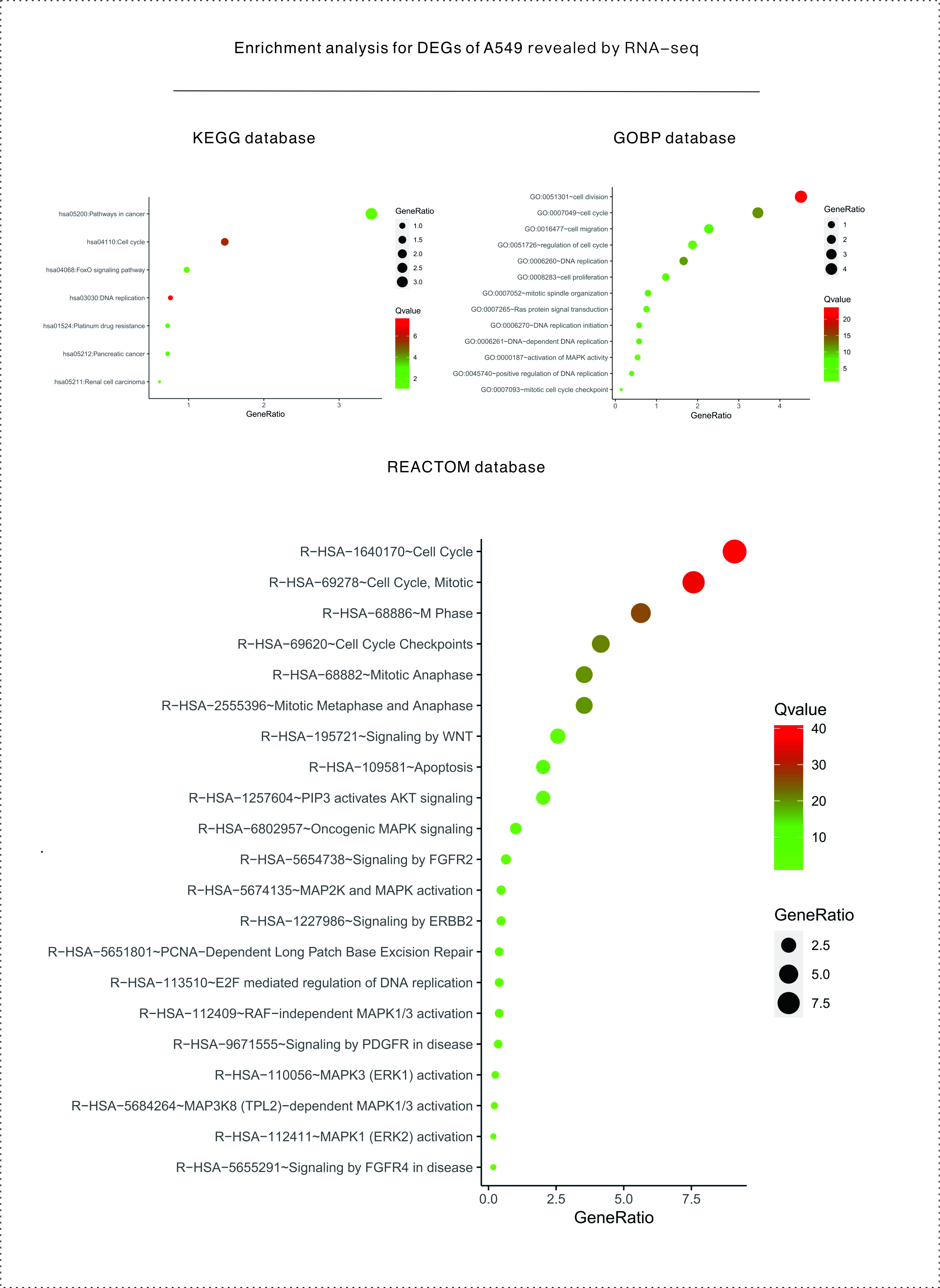

Supplement: Supplementary file 3 — Additional file 2 Figure S2. Enrichment analysis based on differentially expressed genes revealed by RNA-seq sequencing of the A549 cell line. [file 12964_2023_1065_MOESM3_ESM.jpg]
