# Supplementary material for: Multiomics analysis revealed the mechanisms related to the enhancement of proliferation, metastasis and EGFR-TKI resistance in EGFR-mutant LUAD with ARID1A deficiency
Source: Cell Commun Signal. 2023 Mar 3;21:48. doi: 10.1186/s12964-023-01065-9 (PMC9985251; doi:10.1186/s12964-023-01065-9)
Supplement: Supplementary file 5 — Additional file 4. Table S1. Antibodies and lentivirus sequences. [file 12964_2023_1065_MOESM5_ESM.doc]

**Table S1. Antibodies and lentivirus sequences**

| **Antibodies** | | | |
| --- | --- | --- | --- |
| **Name** | **Manufacturer** | **Number** | **Dilution rate** |
| ARID1A | Abcam | ab182560 | 1:500 (IHC); 1:1000 (WB) |
| phosphorylated-EGFR | Cell Signaling Technology | 3777 | 1:1000 |
| phosphorylated-AKT | Cell Signaling Technology | 4060 | 1:2000 |
| phosphorylated-MAPK | Cell Signaling Technology | 4695 | 1:1000 |
| phosphorylated-HER2 | Abcam | ab108371 | 1:500 |
| phosphorylated-HER3 | Abcam | ab133445 | 1:1000 |
| phosphorylated-HER4 | Abcam | Ab76132 | 1:1000 |
| β-actin | Sigma-Aldrich | A3854 | 1:5000 |
| **Lentivirus sequences (5’-3’)** | | | |
| sh-ARID1A | GTTGATGAACTCATTGGTT | | |
| Vector control | TTCTCCGAACGTGTCACGT | | |
